# Nor climate nor human impact factors: Chytrid infection shapes the skin bacterial communities of an endemic amphibian in a biodiversity hotspot

**DOI:** 10.1002/ece3.11249

**Published:** 2024-04-08

**Authors:** Leonardo D. Bacigalupe, Jaiber J. Solano‐Iguaran, Ana V. Longo, Juan D. Gaitán‐Espitia, Andrés Valenzuela‐Sánchez, Mario Alvarado‐Rybak, Claudio Azat

**Affiliations:** ^1^ Instituto de Ciencias Ambientales y Evolutivas Universidad Austral de Chile Valdivia Chile; ^2^ Departamento de Salud Hidrobiológica Instituto de Fomento Pesquero Puerto Montt Chile; ^3^ Department of Biology University of Florida Gainesville Florida USA; ^4^ School of Biological Sciences and the SWIRE Institute of Marine Science The University of Hong Kong Hong Kong SAR China; ^5^ Institute of Zoology, Zoological Society of London London UK; ^6^ ONG Ranita de Darwin Santiago Chile; ^7^ Núcleo de Ciencias Aplicadas en Ciencias Veterinarias y Agronómicas Universidad de Las Américas Santiago Chile; ^8^ Sustainability Research Centre & PhD in Conservation Medicine Universidad Andres Bello Santiago Chile

**Keywords:** 16S rRNA sequencing, amphibian skin, Chilean biodiversity hotspot, chytrid fungus, microbial diversity, microbiome

## Abstract

The bacterial communities of the amphibian skin (i.e., the bacteriome) are critical to the host's innate immune system. However, it is unclear how different drivers can alter this function by modulating the bacteriome's structure. Our aim was to assess the extent to which different host attributes and extrinsic factors influence the structure of the bacterial communities of the skin. Skin bacterial diversity was examined in 148 individuals of the four‐eyed frog (*Pleurodema thaul*) from 16 localities spanning almost 1800 km in latitude. The richness and beta diversity of bacterial families and the richness and abundance of *Bd*‐inhibitory bacterial genera were used to describe their structure. Predictors associated with the host (developmental stage, genetic lineage, individual *Batrachochytrium dendrobatidis* [*Bd*] infection status) and the landscape (current climate, degree of anthropogenic disturbance) were used in the statistical modeling in an information theoretical approach. *Bd* infection and host developmental stage were the only predictors affecting bacteriome richness, with *Bd*+ individuals and postmetamorphic stages (adults and juveniles) having higher richness than *Bd*− ones and tadpoles. High diversity in *Bd*+ individuals is not driven by bacterial genera with known anti‐*Bd* properties. Beta diversity was not affected by *Bd* infection and was mainly a consequence of bacterial family turnover rather than nestedness. Finally, for those bacterial genera known to have inhibitory effects on chytrid*, Bd+* individuals had a slightly higher diversity than *Bd*− ones*.* Our study confirms an association between *Bd* infection and the host developmental stage with the bacterial communities of the skin of *P. thaul.* Unexpectedly, macroclimate and human impact factors do not seem to play a role in shaping the amphibian skin microbiome. Our study exemplifies that focusing on a single host–parasite system over a large geographic scale can provide essential insights into the factors driving host–parasite–bacteriome interactions.

## Graphical abstract

We assessed the extent to which different host attributes (genetic lineage, age, *Bd* infection) and extrinsic factors (climate, human impact factors) influence the structure of the skin microbiome of the four‐eyed frog (*Pleurodema thaul*) along a latitudinal gradient. *Bd* infection and host developmental stage were the only predictors affecting microbiome richness. Macroclimate and human impact factors do not seem to play a role in shaping the amphibian skin microbiome.

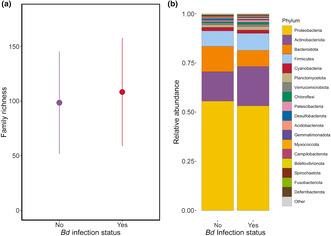

## 1 INTRODUCTION

Multicellular organisms are host to symbiotic microbial communities, which significantly impact several aspects of their health and fitness (Peixoto et al., 2022; Ross et al., 2019). In amphibians, the bacterial communities of the skin (i.e., the bacteriome) are a vital component of the innate immune system and provide a first and critical line of defense against pathogens (Harris et al., 2009; Rebollar et al., 2020). Such beneficial functional effect is determined by the richness and composition of the bacteriome, which results from the interplay of ecological and evolutionary processes acting at different temporal and spatial scales on both the amphibian host and the symbiotic microbial communities (Muletz‐Wolz et al., 2018). Some of the essential processes shaping the structure of the bacterial communities of the skin are related to specific characteristics of the host, the locality, landscape, and geographic factors (Rebollar et al., 2020).

Despite the increased number of studies documenting intra‐ and interspecific variation in the structure of amphibian skin bacteriome, there are still significant limitations (e.g., spatial, temporal, and species coverage) that prevent us from having a thorough understanding of how those different processes (and their potential interactions) underpin its variation, which is highly context‐dependent. For instance, while intraspecific variation in bacteriome structure is usually low (Prado‐Irwin et al., 2017), variation at the interspecific level has been observed even for syntopic species (i.e., taxa that overlap both spatially and temporally) (Belden et al., 2015; Bletz et al., 2017). In addition, several studies have shown the impact of climatic conditions on the richness and composition of the bacterial communities of the skin across various geographic scales, from regional to global (Kueneman et al., 2019; Ruthsatz et al., 2020; Woodhams et al., 2020). Furthermore, the impact of these factors can be exacerbated by the adverse effects of contemporary anthropogenic drivers of global change, altering microbial communities' richness and phylogenetic diversity (Becker et al., 2017). Ecological interactions also influence the bacteriome, and in the context of the current amphibian decline crisis, it may have a crucial role in providing protection against *Batrachochytrium dendrobatidis* (*Bd*), the fungal pathogen causing the emerging infectious disease, amphibian chytridiomycosis. For example, the bacterial communities of the skin can modulate and contribute to host immunity by directly inhibiting *Bd* growth (e.g., Bletz et al., 2013, 2017; Woodhams et al., 2015). Furthermore, the structure of the skin bacteriome has been associated with the balance between epizootic and enzootic *Bd* infection dynamics in wild populations as outbreaks leave signals of disturbance in the microbial community composition (Bates et al., 2022; Jani et al., 2021). It is also known that *Bd* infection can modify the structure of the bacterial communities of the skin, indicating a potential ecological co‐regulation (Jani et al., 2017; Jani & Briggs, 2018; Longo et al., 2015; Rebollar et al., 2016). This bidirectional relationship further highlights the inherent complexities of the host, pathogen, and bacteriome triangle, which, in addition, might be influenced by environmental factors (Greenspan et al., 2022; Ruthsatz et al., 2020). Although untangling these complex relationships might be difficult in a non‐experimental and non‐manipulative setting, studies in a single host species over a broad geographic scale offer an alternative to tease apart the mechanisms by which the bacterial communities of the amphibian skin are modulated by the individual or interactive influence of host‐specific characteristics, the locality, and geographic factors.

Here, we evaluate macro‐geographic patterns of the bacterial communities of the skin using the four‐eyed frog (*Pleurodema thaul*) as our host study model. This species has an extensive latitudinal distributional range (>2500 km, 27° S–45° S) (Vidal & Díaz‐Páez, 2012), and thus, it covers an extensive number of biomes, climates, and gradients of anthropogenic influence within the Chilean biodiversity hotspot. Furthermore, *Bd* infection in this species has been extensively studied, and its wide range includes local populations with different infection status and prevalence (Bacigalupe et al., 2017). Therefore, *P. thaul* is an excellent model to disentangle the influence of multiple factors that could shape the amphibian skin bacteriome. Specifically, we assessed whether the structure of the bacterial communities of the skin is affected by factors associated with the host (i.e., developmental stage and genetic lineage, individual *Bd* infection status) and the landscape (i.e., current climate and degree of anthropogenic disturbance). Moreover, by using high‐resolution data, we were able to evaluate potential synergistic effects among different factors on the skin bacteriome at the landscape level, which has proved critical to explaining patterns of *Bd* prevalence in the country (Bacigalupe et al., 2017). We have the following predictions regarding overall trends. First, we expect a negative association between the richness of the bacterial communities of the skin at the family level and the intensity of anthropogenic drivers, as the latter is likely to favor those taxa that can thrive in perturbed habitats (e.g., Costa et al., 2016). Second, given the broad latitudinal gradient covered and, therefore, the concomitant cline in environmental temperature, we expect the beta diversity of the skin bacteriome to be explained mainly by the component of turnover rather than nestedness. We predict a high turnover of bacterial families mainly as a consequence of spatial differences in climate, as different families would have contrasting thermal growth optima. In contrast, the beta diversity component of nestedness predicts that skin bacteriomes represent different subsets of bacterial families from sites characterized by high diversity. Third, as more variable thermal environments may favor families with different thermal growth optima, we expect an increased bacterial richness in locations with higher temperature seasonality.

## 2 MATERIALS AND METHODS

### 2.1 Individual sampling and *Bd* infection data

The amphibian skin microbiome was obtained from tadpoles, juveniles, and adults of *P. thaul* from 16 localities spanning almost 1800 km in latitude (Table 1). Complete details on the swabbing and *Bd* diagnostic analysis can be found in Alvarado‐Rybak et al. (2021). Briefly, the extracted DNA was diluted at a ratio of 1:10 in double‐distilled water and subjected to analysis using a quantitative real‐time PCR Taqman assay (qPCR). The primers utilized were specific for the ITS‐1/5.8S ribosomal DNA region of *Bd*, following the methodology outlined by Soto‐Azat et al. (2013). To mitigate PCR inhibition, bovine serum albumin (BSA) was incorporated into the Taqman mastermix, as suggested by Garland et al. (2010). Each sample underwent diagnostic assays in duplicate, and within each PCR plate, standards with known zoospore concentrations were included, alongside negative controls. The structure of the skin microbiome was recovered from the same DNA extractions of skin swabs used for previous *Bd* detection analyses.

**TABLE 1:** Sampling locations for bacteriome analyses of four‐eyed frog (*Pleurodema thaul*).

Locality	Lineage	Latitude	Longitude	*N*	# Positives	Year
Carrera Pinto	North	−27.11	−69.91	13	0	2017
Villa Las Palmeras	North	−28.53	−70.92	14	2	2017
Río Elqui	North	−29.89	−71.26	2	2	2015
Limari	North	−30.67	−71.52	8	0	2016
Illapel	North	−31.62	−71.14	11	3	2017
Villa Alemana	North	−33.04	−71.37	5	4	2016
Quebrada Escobares	North	−33.09	−71.29	11	10	2011
Parque Safari	North	−34.19	−70.80	5	0	2017
Laguna Torca	North	−34.78	−72.04	11	8	2008
Buche	North	−34.98	−71.73	9	0	2016
Río Mataquito	North	−35.05	−71.74	9	5	2016
Hualqui	Centre	−37.02	−72.97	12	7	2008
Puente Santa Helena	Centre	−37.70	−72.60	14	8	2017
Boroa	Centre	−39.29	−73.10	10	3	2009
Chaihuin	Centre	−39.97	−73.57	3	0	2017
La Vara	Centre	−41.43	−72.89	11	9	2017

*Note*: Host lineages according to Barria et al. (2020). *N* = number of samples used for bacteriome analyses. # Positives = Number of *Bd*+ frogs in the corresponding *N*. Year = year of collection of bacteriome samples used in this study.

### 2.2 Amphibian skin microbiome

From a total of 398 samples, we randomly selected the DNA extraction of 195 skin swabs (*N*_adults_ = 108, *N*_juveniles_ = 69, *N*_tadpoles_ = 18) to quantify bacterial diversity in the 16 *P. thaul* studied populations. The number of individuals sampled for each developmental stage and population is presented in Table S1. Bacterial DNA was extracted using the PowerSoil DNA isolation kit (MoBio) in accordance with the manufacturer's guidelines, except for the elution step, which involved 25 μL of Milli‐Q water. Next‐generation sequencing libraries were prepared at the AUSTRAL‐omics core research facilities of the Universidad Austral de Chile (Valdivia, Chile). The V3‐V4 hypervariable region of the 16S rRNA gene was targeted for amplification and sequencing, utilizing specific primers selected from Klindworth et al. (2013). Library construction followed the Illumina protocol for 16S Metagenomic Sequencing Library, employing 341F (5′CCTACGGGNGGCWGCAG3′) and 805R (5′CTACHVGGGTATCTAATCC3′) primers with attached Illumina index adapters, as detailed by Bourlat et al. (2016). In addition to the 16S primers, the sequencing design incorporated elements from Fadrosh et al. (2014), featuring a linker sequence optimized for Illumina sequencing, index sequences, and a heterogeneity spacer. Amplicon sequencing was carried out using 300 bp paired‐end reads on an Illumina MiSeq sequencer (Illumina, San Diego, CA).

### 2.3 Bioinformatic data analyses

Initial quality filtering was conducted using the BBTools package with the bbduk.sh script (Bushnell & Rood, 2018) removing reads shorter than 100 bp (min. length = 100) and with average quality below 20 (min. avg. quality = 20). Filtered raw data were demultiplexed with *cutadapt V 2.0* (Martin, 2011) allowing no more than 1 mismatch in barcodes sequences (‐e 0.1). Demultiplexed reads were then used to infer the amplicon sequence variants (ASVs) with the R package *DADA2* (Callahan et al., 2016), following the pipeline recommended by the authors. Chimeric sequences were identified and removed with the function “removeBimeraDenovo” DADA2. Then, ASVs were mapped to the SILVA138 bacterial 16S rRNA database (Quast et al., 2013) with the RDP naïve Bayesian classifier (Wang et al., 2007) to assign bacterial taxonomy. The resulting filtered alignment was used to infer an approximate maximum‐likelihood phylogenetic tree constructed in FastTree (Price et al., 2009). ASVs were filtered and removed if sequences were identified as mitochondria or chloroplast, and if they had total abundances lower than 50 reads. The number of reads varied from 206 to 499,487. To standardize the samples' depth, rarefaction was applied to 3163 reads per sample, resulting in the retention of 148 samples (*N*_adults_ = 87, *N*_juveniles_ = 45, *N*_tadpoles_ = 16) (Table S1).

### 2.4 Predictors of bacteriome structure

We used eight potential predictors of microbiome structure in the statistical modeling. At the individual level, these include *Bd* infection status (positive or negative), host developmental stage (tadpole, juvenile, adult), and host genetic lineage. Regarding the latter, Barria et al. (2020) determined that three major clades comprised *P. thaul*'s evolutionary diversity across its distributional range (north, central, and south). However, the southern clade was not represented in our sampled individuals. At the landscape level, we included annual mean temperature, temperature seasonality, and annual precipitation as well as human footprint, a composite index that characterizes human influence on the land‐based on accessibility, anthropogenic land use, population density, and infrastructure (Bacigalupe et al., 2019; Sanderson et al., 2002). Climate variables for each geo‐referenced locality were obtained from WorldClim with a resolution of 30 arc seconds (Hijmans et al., 2005) using ArcGIS 10.1. Finally, as samples were independently collected in different years (see Table 1), we also included year as a predictor to account for potential temporal structuring of the data.

### 2.5 Diversity metrics of bacteriome composition and structure

#### 2.5.1 Bacterial family richness

Generalized linear mixed models with Poisson error structure in an information‐theoretic framework (Burnham & Anderson, 2002) were employed to contrast the adequacy of 10 working hypotheses explaining the richness of bacterial families while considering the locality of the sampled individuals as a random factor. The number of candidate models was kept to a minimum to reduce the likelihood of spurious results (Burnham & Anderson, 2002; Lukacs et al., 2010). Furthermore, given our relatively low sample size, all models except one included a single predictor. We evaluated the following models: (i) null model (without predictors), (ii) *Bd* infection status, (iii) host developmental stage, (iv) host genetic lineage, (v) annual mean temperature, (vi) temperature seasonality, (vii) annual precipitation, (viii) human footprint, (ix) annual mean temperature in interaction with human footprint, and (x) year. Each evaluated model represents either a specific hypothesis proposed here (i.e., models vi and viii) or that previous research has shown to be significant (i.e., models ii, iii, iv, v, vii, and ix, see Section 4) or represents a potential statistical issue (i.e., models i and x). We employed Akaike's information criterion (AIC), which takes into consideration the likelihood of each model while penalizing for the number of parameters (*K*) to obtain the best‐ranked model, and Akaike's weights (*wi*) to quantify the relative support of each model in the set (Burnham & Anderson, 2002; Turkheimer et al., 2003). All analyses and graphics were implemented in R 3.6.3 (R Development Core Team, 2020) using packages *ggplot2* (Wickham, 2016), *lme4* (Bates et al., 2015), *MuMIn* (Barton, 2022), *phyloseq* (McMurdie & Holmes, 2013), and *vegan* (Oksanen et al., 2022).

#### 2.5.2 Beta diversity

The bacterial composition at the family level was partitioned into the two components of beta diversity: turnover (i.e., *β*_SIM_, family replacement) and nestedness (i.e., *β*_SNE_, family loss) using the Sørensen dissimilarity index (i.e., *β*_SOR_, Baselga, 2010). We used a resampling procedure (*N* = 10,000) of five localities (out of the 16) each time and recalculated the three dissimilarity indexes to obtain a distribution of each one. From each distribution, mean and standard deviation values were calculated. To understand the impact of *Bd* on bacterial family composition, we also evaluated *β* diversity separately for *Bd*+ and *Bd*− individuals using a similar resampling procedure. Total *β* diversity and its components were compared between infected and non‐infected individuals. *p*‐Values were obtained as the number of times a *β* coefficient was higher in one group of individuals versus the other divided by the number of times each index was calculated (i.e., 10,000). Analyses were carried out using the R package *betapart* (Baselga et al., 2022).

#### 2.5.3 Richness and abundance of *Bd*‐inhibitory bacterial at the genus level

We quantified changes in the richness and abundance of bacterial genera (*N* = 52) between *Bd*+ and *Bd*− individuals for those isolates known to have inhibitory effects on *Bd* (Woodhams et al., 2015). Given the constraints associated with accurately assigning bacterial taxa at the species level using the 16S V3‐V4 regions (Jeong et al., 2021), our analysis adopted the genus level within the taxonomic hierarchy for a more reliable assessment. In particular, we used diversity profiles based on Hill numbers (i.e., *q*) using the framework described by Chao et al. (2020) to calculate overall taxa (i.e., bacterial genus for this study) diversity (*q* = 0), Shannon diversity (*q* = 1), and Simpson diversity (*q* = 2). Richness counts all taxa equally without considering their frequencies, and thus, it is most sensitive to rare taxa. Shannon diversity considers the relative abundance of each taxon and thus can be considered as the effective number of frequent taxa. Simpson diversity is highly sensitive to those dominant taxa in high frequencies. We applied the interpolation and extrapolation procedure of Colwell et al. (2012) and Chao and Jost (2012), as implemented in the R package iNEXT‐4‐steps (https://github.com/AnneChao/iNEXT.4steps) to calculate Hill numbers of orders *q* = 0, 1, and 2 and their corresponding bootstrapped (50 runs) 95% confidence intervals for comparison between our treatments (i.e., *Bd*+ and *Bd*− individuals) (Chao et al., 2020). Before comparing diversities between treatments, we first quantified sample completeness for each of the three diversity measures (Chao et al., 2020). As sample completeness was total (i.e., 100%) for all three orders *q* = 0, 1, and 2, further standardization was not required (Chao et al., 2020) (see below).

## 3 RESULTS

### 3.1 Alpha diversity of bacterial families is mostly explained by chytrid infection

**FIGURE 1:**
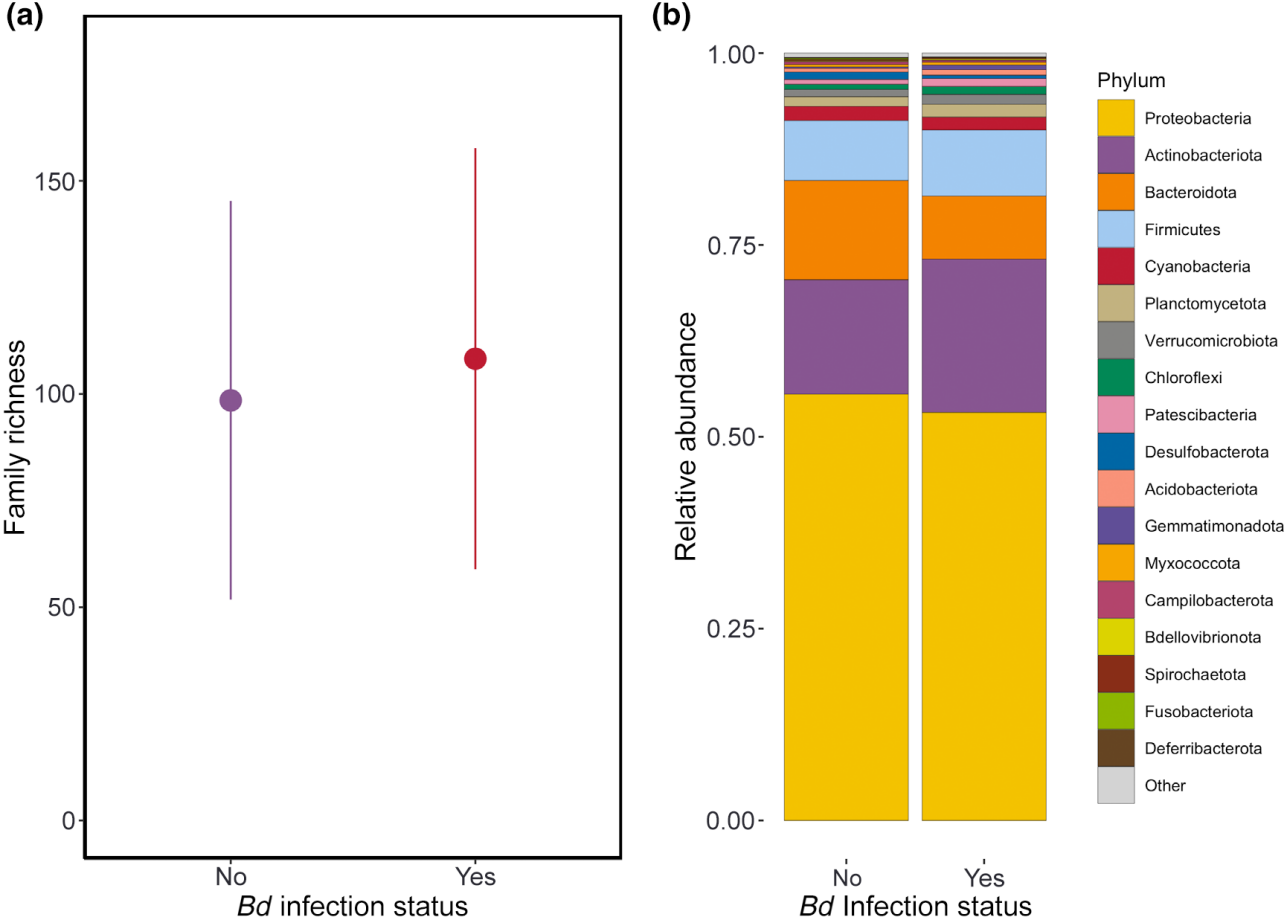
(a) Mean family richness ± SD for infected (Yes) and non‐infected (No) individuals with *Batrachochytrium dendrobatidis*. (b) Relative abundance of amplicon sequence variants by major phyla after rarefaction at 3163 reads per individual. Note that phylum data are presented for descriptive purposes only, as analyses were conducted at the family level.

The model selection procedure indicated that two models, one using *Bd* infection status and the other developmental stage as a predictor, were notably more parsimonious than the null model (i.e., the ΔAIC_c_ between the null model and each of these models was >19; Table 2). The model with annual mean precipitation had a similar AIC_c_ value to the null model (ΔAIC_c_ < 1). All the other candidate models were worse ranked than the null model. A likelihood ratio test between the null model and each of the three best‐ranked models confirmed that both *Bd* and developmental stage, but not annual mean precipitation, had a statistically significant effect on bacterial family richness (*Bd*: *χ* ^2^_[1]_ = 39.68, *p* < .01; developmental stage: *χ* ^2^_[1]_ = 23.97, *p* < .01; precipitation: *χ* ^2^_[1]_ = 2.99, *p* = .08). In the best‐ranked model (i.e., *Bd* infection status as predictor), *Bd*+ individuals showed higher bacterial family richness than *Bd*− ones (Figure 1a). The phylum Proteobacteria comprised, after rarefaction, more than half the classified sequences, dominating microbial communities in both *Bd*+ (i.e., 55.6%) and *Bd*− individuals (i.e., 53.2%) (Figure 1b). The remaining sequences belonged primarily to Actinobacteriota (*Bd*+ = 14.9%; *Bd*− = 20%), Bacteroidota (*Bd*+ = 12.9%; *Bd*− = 8.2%), and Firmicutes (*Bd*+ = 7.8%; *Bd*− = 8.6%). The second‐ranked model showed that tadpoles had lower bacterial family richness than adults and juveniles (Figure S1a). This result is not an artifact of the unbalanced sampling for the different developmental stages at every location (see Table S1): A rarefaction analysis indicated that tadpoles and juveniles had higher bacterial diversity than adults (Figure S2). The phylum composition by developmental stage followed a similar pattern to the one in *Bd*+ and *Bd*− individuals, with Proteobacteria encompassing more than half the sequences, followed by Actinobacteriota, Bacteroidota, and Firmicutes (Figure S1b). Although the model with annual mean precipitation also ranked better than the null one, the 95% CI for its coefficient overlaps zero (*b* = −0.121, 95% CI: −0.261 to 0.018). The rest of the models, including individual (genetic lineage) or landscape (climate and human footprint) predictors, had very low support (Table 2). Finally, year was also not supported as a predictor, which indicates that even if samples were collected in different years, the sampling had no temporal effects.

**TABLE 2:** Models accounting for bacteriome richness at the family level in *Pleurodema thaul.*

Model^a^	*K*	AIC_c_	ΔAIC_c_	LogLik	*w*_*i*_
*Bd*	3	3515.1	0	−1754.48	1
Developmental stage	4	3532.9	17.82	−1762.33	0
PCP	3	3551.8	36.69	−1772.82	0
Null (no predictors)	2	3552.7	37.59	−1774.32	0
Genetic lineage	3	3553.8	38.67	−1773.82	0
*T*	3	3553.9	38.78	−1773.87	0
*T*_sd_	3	3554.7	39.61	−1774.23	0
HFI	3	3554.8	39.63	−1774.30	0
Year	3	3554.8	39.66	−1774.31	0
*T* * HFI	5	3555.1	39.97	−1772.34	0

*Note*: *Bd* = individual infection status, *T* = annual mean temperature, *T*_sd_ = temperature seasonality, PCP = mean annual precipitation, HFI = human footprint index, *K* = number of parameters, AIC_c_ = AIC values corrected for small sample sizes, LogLik = Log likelihood. *w*_*i*_ = Akaike's weights.

^a^ All models included locality as a random factor and are sorted accordingly with their AIC_c_.

### 3.2 Beta diversity is characterized by bacterial family turnover

**FIGURE 2:**
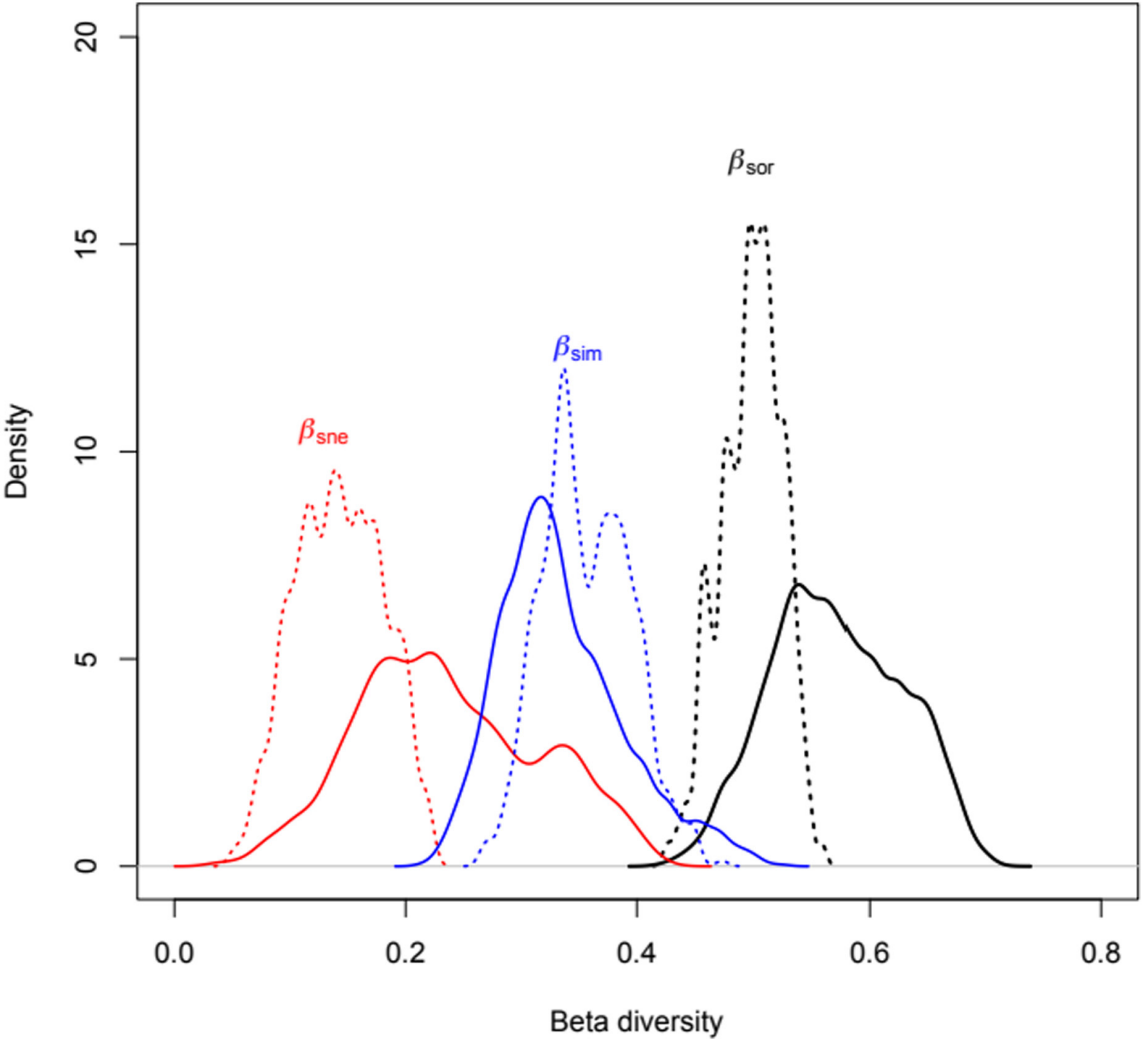
Partition of Sørensen beta diversity (*β*_SOR_) in turnover (*β*_SIM_) and nestedness (*β*_SNE_) at the family level in four‐eyed frogs (*Pleurodema thaul*) that tested positive (solid line) or negative (dashed line) for the presence of *Batrachochytrium dendrobatidis*. Distributions were obtained using 10,000 samples of five locations each (see Section 2 for further details). Beta diversity indexes did not differ as a result of *Bd* infection.

Overall *β* diversity was *β*_SOR_ = 0.630 ± 0.015 and was mostly a consequence of family turnover (*β*_SIM_ = 0.471 ± 0.016, *β*_SNE_ = 0.159 ± 0.019). In addition, *β* diversity in *Bd*− individuals (*β*_SOR_ = 0.572 ± 0.056) was not higher than *β* diversity in *Bd*+ ones (*β*_SOR_ = 0.499 ± 0.026) (*p* = .89) (Figure 2). Most of this diversity is accounted for by bacterial family turnover, which also was not higher in non‐infected individuals (*Bd*+: *β*_SIM_ = 0.357 ± 0.038; *Bd*−: *β*_SIM_ = 0.335 ± 0.054) (*p* = .34) (Figure 2). Nestedness represented a small fraction of all *β* diversity and was not statistically smaller in *Bd*+ individuals (*β*_SNE_ = 0.143 ± 0.038) with respect to *Bd*− ones (*β*_SNE_ = 0.237 ± 0.078) (*p* = .86) (Figure 2).

### 3.3 Diversity of *Bd*−inhibitory bacterial genera varied between infection status

**FIGURE 3:**
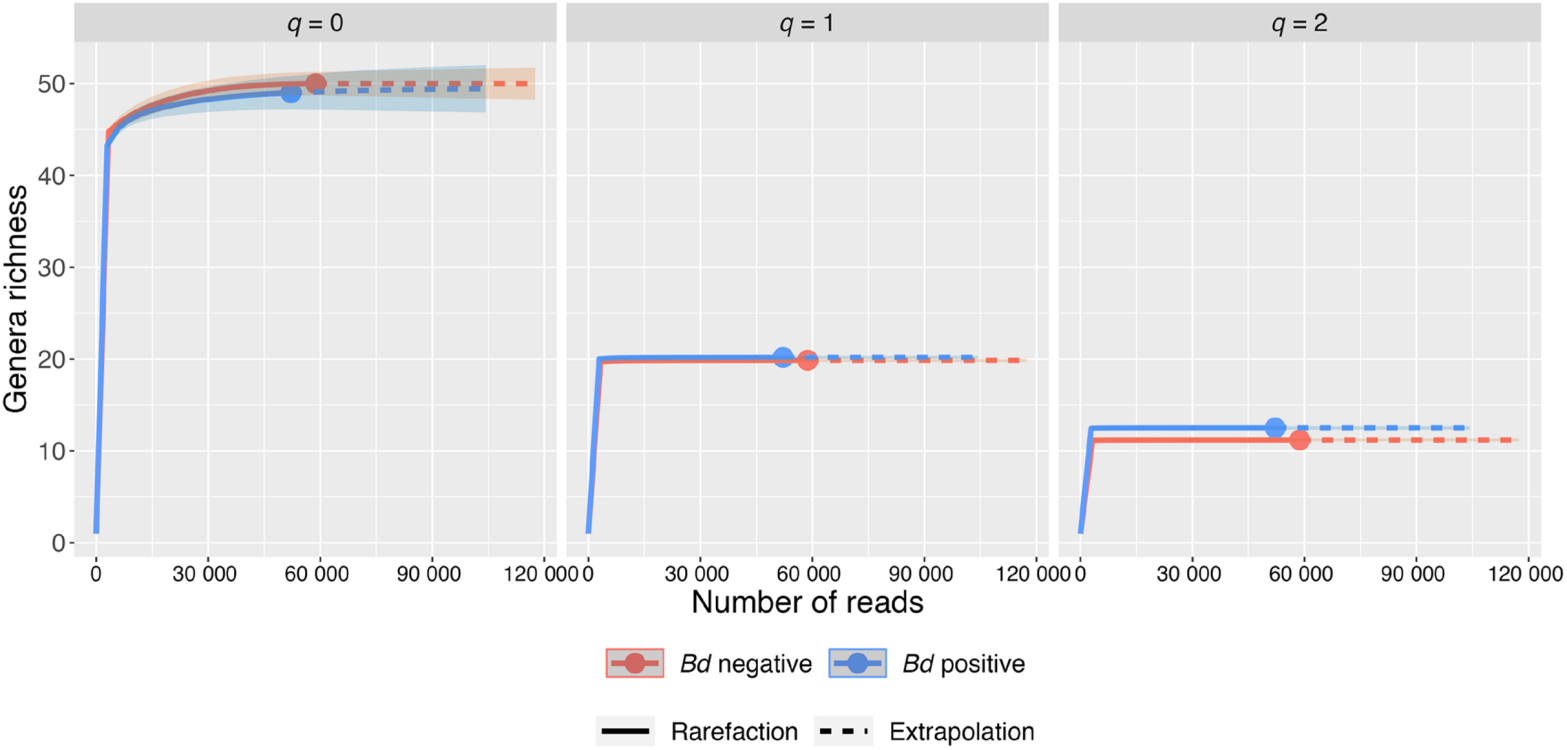
Sample‐size‐based rarefaction (solid lines) and extrapolation curves (dashed lines) up to double the reference sample size for order *q* = 0 (Richness), 1 (Shannon diversity) and 2 (Simpson diversity) for *Bd*− and *Bd*+ individuals. Solid dots denote observed diversity. All curves have a very narrow shaded area which denotes 95% confidence bands obtained by bootstrapping with 50 replications.

The estimated sample completeness for *q* = 0, 1, and 2, for both *Bd*+ and *Bd*− individuals, was 100%. This means that, irrespective of *Bd* infection status, there are no undetected genera (*q* = 0); the detected genera cover 100% of the reads (*q* = 1) and 100% of the reads, if the focus was solely on highly abundant genera (*q* = 2). Rarefaction and extrapolation sampling curves for *q* = 0, 1, and 2 stabilized for both *Bd*+ and *Bd*− individuals (Figure 3), indicating that the asymptotic estimates are reliable. Our results indicate that the same bacterial genera with known inhibitory effects on *Bd* were shared between *Bd*+ and *Bd*− individuals (Figure 3, Figure S3). Regarding abundant genera (*q* = 1), *Bd*− individuals are equally diverse as *Bd*+ ones, as indicated by their overlapping 95% CI (Figure 3). When considering highly abundant genera (*q* = 2), *Bd*+ individuals have are slightly more diverse (*N* = 1.31) than non‐infected ones (Figure 3). Finally, Pielou's evenness (i.e., *q* = 0) among bacterial genera was almost identical between *Bd*− (0.76) and *Bd*+ (0.77) frogs.

## 4 DISCUSSION

Previous landscape‐level bacteriome studies in amphibians included many host species across latitudinal gradients, which can potentially introduce confounding factors related to differences in host/bacteriome co‐evolutionary trajectories. Our study leverages the widespread distribution of a single species to disentangle the effect of several factors shaping the bacterial communities of the amphibian skin. Our results show that (i) *Bd* infection status and host developmental stage were the only predictors affecting bacterial (family) richness, (ii) β diversity is primarily a consequence of bacterial family turnover instead of nestedness, and it is not affected by *Bd* infection, and (iii) for those bacterial genera that are known to have inhibitory effects on *Bd, Bd*+ individuals are slightly more diverse for highly abundant genera than *Bd*+ ones.

### 4.1 Bacterial communities are homogeneous across genetic lineages but not host age

Contrary to what others have found (e.g., Ellison et al., 2018; Jani & Briggs, 2018), our results showed that the richness of bacterial families was not affected by the genetic lineage of the host, suggesting that the structure of the bacteriome is conserved at these shallow phylogenetic scales; as it has been previously recognized (e.g., Buttimer et al., 2021; Prado‐Irwin et al., 2017). Although our analyses are based on a relatively low sample size (i.e., 16 statistically independent data points) unevenly distributed in two lineages out of three (Barria et al., 2020), the model selection procedure did not find any support for this predictor.

Furthermore, it has been shown that the bacterial communities of the skin change in diversity and composition across host developmental stages (Griffiths et al., 2018; Prest et al., 2018), probably because of the major modifications and increase in complexity of the immune system during metamorphosis (Woodhams et al., 2020). Our results show that bacterial family richness was higher in adult and juvenile individuals than in tadpoles (Figure S1). Nevertheless, it remains to be assessed whether this difference in richness is a consequence of the immune system's reorganization associated with the metamorphosis. For example, Longo et al. (2015) show that juveniles of *Eleutherodactylus coqui*, a species with direct development (i.e., sub‐adults hatch from eggs), exhibited higher bacterial richness in comparison to adults, which they attributed to the fact that the juvenile stage is the most susceptible to chytridiomycosis (Langhammer et al., 2014; Longo et al., 2010).

### 4.2 Climate and anthropogenic factors do not affect the bacteriome alpha diversity

Several studies have shown that climatic factors, over regional or global geographic scales, affect the structure of the bacterial communities of the skin (e.g., Kueneman et al., 2019; Ruthsatz et al., 2020; Woodhams et al., 2020). Our results do not support this finding, as all five evaluated models, including either a climatic or anthropogenic factor, showed low support (Table 2). Although the model with annual mean precipitation ranked better than the null one, the 95% CI for its coefficient overlaps zero. At present, we are not able to rule out that this result might be more a statistical issue (given our low sample size) than a real effect of precipitation on the bacteriome richness. In any case, the negative trend observed in bacterial richness with annual mean precipitation does not agree with what has been found on a global (Kueneman et al., 2019) or regional (Ruthsatz et al., 2020) scale. Kueneman et al. (2019) found that the diversity of skin bacteria on a global scale showed a consistent association (negative) with temperature‐related variables, while Ruthsatz et al. (2020) showed that precipitation is positively associated with bacterial richness in the Brazilian Atlantic Forest.

Furthermore, we predicted that more variable thermal environments would favor more thermal specialization, and thus, a positive relationship between bacterial family richness and temperature seasonality was expected. Our results indicate that such thermal specialization does not occur, suggesting that a similar number of families are found along the latitudinal gradient. Moreover, as β diversity results mostly from family turnover (Figure 2), overall, it might indicate that bacterial families with different thermal optima are being replaced from north to south so that the functional properties of the community are conserved in space and time.

Contrary to our predictions, bacterial family richness was not affected by anthropogenic influence, as the model with human footprint also showed barely any support. These results highlight that anthropogenic change might not affect the capacity of hosts to recruit bacterial taxa, especially if the members of the skin bacteriome are decoupled from species pools in the environment. In addition, as human footprint does not follow a latitudinal gradient (results not shown), the replacement of families shown by the β diversity results indicates that replacement is not caused by bacterial families that thrive under human influence. It is important to mention, though, that *P. thaul* is a species that, in general, can tolerate and even thrive in habitats with anthropogenic influence (Vidal & Díaz‐Páez, 2012), and therefore, human impact factors might be more noticeable on a more vulnerable species and its associated skin bacteriome.

### 4.3 Bacteriome diversity and *Bd* infection

The interaction between the bacterial communities of the amphibian skin and *Bd* is far from straightforward (Jiménez & Sommer, 2017). Although it is known that these communities are an essential defense barrier against pathogens (e.g., Bletz et al., 2017; Woodhams et al., 2015), *Bd* infection can also disturb their structure (e.g., Jani & Briggs, 2018). Our results show that *Bd*+ individuals had about 10 bacterial families more than non‐infected ones (Figure 1).

Although at first this could be considered as the result of an active recruiting of beneficial microorganisms, our results also show that both *Bd*+ and *Bd*− individuals carry all bacterial genera that are known to have inhibitory effects on *Bd* (*q* = 0, Figure 3, Figure S3). Another potential explanation is that those additional bacterial families in *Bd*+ individuals are indeed beneficial in terms of their function against *Bd*. However, at the moment, their function is unknown without further experimental work. A more plausible explanation is that those additional families come from the large pool of shared families between *Bd*+ and *Bd*− individuals and, thus, are not being selected for any anti‐*Bd* properties. In particular, infected and non‐infected individuals share 391 (79%) bacterial families out of a total of 495, while *Bd*+ individuals only have 8.5% (*N* = 42) and *Bd*− 12.5% (*N* = 62) exclusive families. We hypothesize that similar to what was found in other species (Longo et al., 2015), *P. thaul*, being generally resistant to developing chytridiomycosis (Alvarado‐Rybak et al., 2021), might experience seasonal *Bd* infections, where throughout the year, individuals alternate their status between being infected, clearing the fungus, and becoming re‐infected again (e.g., Longo et al., 2015). Thus, it is possible that in those periods of re‐infection, *Bd*+ individuals may recruit beneficial bacterial families to clear *Bd*. As families are mostly replaced along the latitudinal gradient regardless of infection status (Figure 2), this recruitment seems relatively consistent along our macro‐geographic scale.

Interestingly, *Bd*+ individuals exhibit slightly more bacterial diversity than *Bd*+ ones regarding highly abundant (*q* = 2) genera known to have anti‐*Bd* effects (Figure 3). Although speculative, this might be why *P. thaul* individuals are not infected in the first place, and why chytridiomycosis‐related mortality in this species or suspected impacts at the population level have not been observed. A more oriented sampling focused on locations and seasons with high prevalence might be required to further enquire into this and disentangle confounding factors (e.g., Longo & Zamudio, 2017a, 2017b). Here, our samples were collected opportunistically on different years, along a 1800 km latitudinal gradient encompassing populations with different *Bd* prevalence, sample sizes, host genetics, climates, and levels of anthropogenic pressure. We maximized the geographical extension of the sampling in order to understand the role of environmental factors (i.e., climate, anthropogenic, and their interactions) in shaping the bacterial communities of the skin.

## 5 CONCLUSIONS

Given that the composition and richness of the bacteriome of the amphibian skin might be affected by climate, anthropogenic influence, host genetic background, *Bd* infection, and other factors (Rebollar et al., 2020), untangling the complex relationships among those factors is essential to inform population management and conservation. Despite all the existing variation in our latitudinal dataset, our study confirms an association between *Bd* infection and host developmental stage with the bacterial communities of the skin of *P. thaul*. The higher bacterial family diversity found in *Bd*+ individuals is not driven by families with known anti‐*Bd* properties. Although the macroclimate and human impact factors seem not to play a role in shaping the amphibian skin bacteriome of our model species (although a potential statistical power problem cannot be discarded), our study highlights the importance of regional geographic scale to really understand the role of environmental factors and the putative role of synergies that might not be obvious or detectable at the global or local scales. Including seasonal and year effects is critical to tease apart temporal from spatial variations on the bacterial communities of the amphibian skin, but just evaluating the role of interactions may shed light on critical future avenues of experimental research. Despite the overall agreement that *Bd* represents a major conservation issue, strategies aimed at mitigating the impact of chytridiomycosis in nature are just beginning (Becker et al., 2023; Garner et al., 2016). Among those, using probiotics to modify the host skin bacteriome holds great potential for targeted disease management (Mueller & Sachs, 2015; Woodhams et al., 2016). However, to start achieving this, it is fundamental to understand more thoroughly the role and potential interactions among the nested‐layered factors (host, microhabitat, landscape) that are known to shape the amphibian skin microbiome as well as the functional aspects of those bacterial families with known anti‐*Bd* properties.

## AUTHOR CONTRIBUTIONS

**Leonardo D. Bacigalupe:** Conceptualization (lead); formal analysis (lead); funding acquisition (lead); visualization (equal); writing – original draft (lead); writing – review and editing (lead). **Jaiber J. Solano‐Iguaran:** Software (lead); visualization (equal); writing – review and editing (equal). **Ana V. Longo:** Formal analysis (supporting); writing – review and editing (equal). **Juan D. Gaitán‐Espitia:** Software (supporting); writing – review and editing (equal). **Andrés Valenzuela‐Sánchez:** Methodology (supporting); writing – review and editing (equal). **Mario Alvarado‐Rybak:** Methodology (supporting); writing – review and editing (equal). **Claudio Azat:** Funding acquisition (equal); methodology (equal); writing – review and editing (equal).

## CONFLICT OF INTEREST STATEMENT

The authors declare no conflict of interest.

## Supporting information


Data S1

## Data Availability

All data and scripts for this study are available at: https://datadryad.org/stash/share/49pfNNiDCs1kg0kAVTdwL6Z02sOodZep5Zv48EwWxNc.
