# Supplementary material for: Nor climate nor human impact factors: Chytrid infection shapes the skin bacterial communities of an endemic amphibian in a biodiversity hotspot
Source: Ecol Evol. 2024 Apr 8;14(4):e11249. doi: 10.1002/ece3.11249 (PMC10999949; doi:10.1002/ece3.11249)

**SUPPLEMENTARY ONLINE MATERIAL**

**Nor climate nor human impact factors: chytrid infection shapes the skin bacterial communities of an endemic amphibian in a biodiversity hotspot**

Leonardo D. Bacigalupe, Jaiber J. Solano-Iguaran, Ana V. Longo, Juan D. Gaitán-Espitia, Andrés Valenzuela-Sánchez, Mario Alvarado-Rybak and Claudio Azat

**Table 1.** Sampling locations for microbiome analyses of four eyed-frog (*Pleurodema thaul*). N = number of samples used for microbiome analyses. # Adults, # Juveniles and # Tadpoles are, respectively, the number of adults, juveniles and tadpoles captured in the corresponding locality.

| Locality | Latitude | Longitude | N | # Adults | # Juveniles | # Tadpoles |
| --- | --- | --- | --- | --- | --- | --- |
| Carrera Pinto | -27.11 | -69.91 | 13 | 10 | 3 | 0 |
| Villa Las Palmeras | -28.53 | -70.92 | 14 | 8 | 6 | 0 |
| Río Elqui | -29.89 | -71.26 | 2 | 1 | 1 | 0 |
| Limari | -30.67 | -71.52 | 8 | 2 | 6 | 0 |
| Illapel | -31.62 | -71.14 | 11 | 4 | 7 | 0 |
| Villa Alemana | -33.04 | -71.37 | 5 | 0 | 5 | 0 |
| Quebrada Escobares | -33.09 | -71.29 | 11 | 11 | 0 | 0 |
| Parque Safari | -34.19 | -70.80 | 5 | 0 | 0 | 5 |
| Laguna Torca | -34.78 | -72.04 | 11 | 11 | 0 | 0 |
| Buche | -34.98 | -71.73 | 9 | 0 | 1 | 8 |
| Río Mataquito | -35.05 | -71.74 | 9 | 6 | 1 | 2 |
| Hualqui | -37.02 | -72.97 | 12 | 11 | 1 | 0 |
| Puente Santa Helena | -37.70 | -72.60 | 14 | 6 | 7 | 1 |
| Boroa | -39.29 | -73.10 | 10 | 10 | 0 | 0 |
| Chaihuin | -39.97 | -73.57 | 3 | 3 | 0 | 0 |
| La Vara | -41.43 | -72.89 | 11 | 4 | 7 | 0 |

**Figure 1**. a) Mean family richness ± SD for tadpoles, juveniles and adults. b) Relative abundance of Amplicon Sequence Variants by major phyla after rarefaction at 3,163 reads per individual.


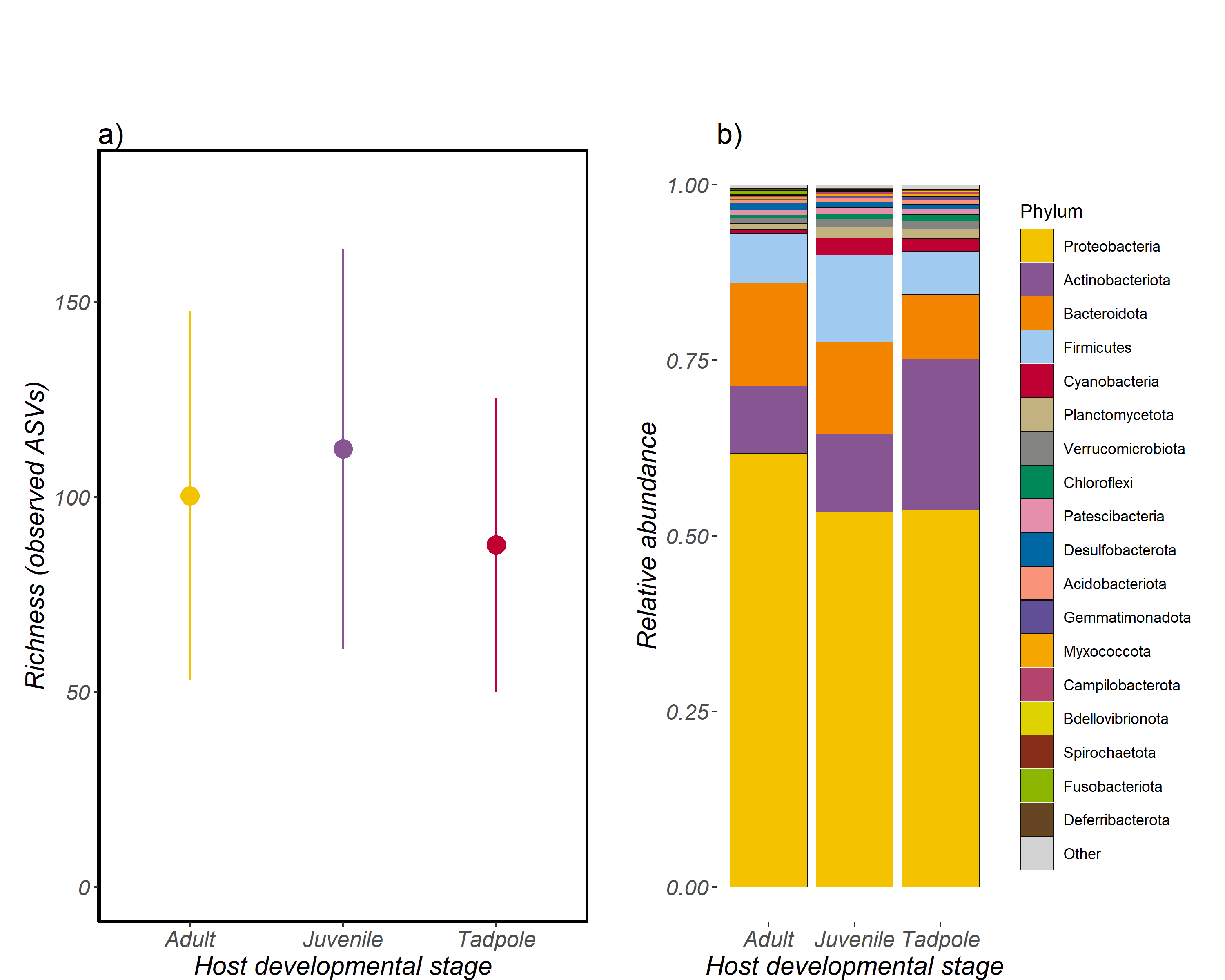


**Figure 2**. Sample-size-based rarefaction (solid lines) and extrapolation curves (dashed lines) up to double the reference sample size for order *q* = 0 (Richness), 1 (Shannon diversity) and 2 (Simpson diversity) for adult, juvenile and tadpoles. Solid dots denote observed diversity. All curves have a very narrow shaded area which denotes 95% confidence bands obtained by bootstrapping with 50 replications.


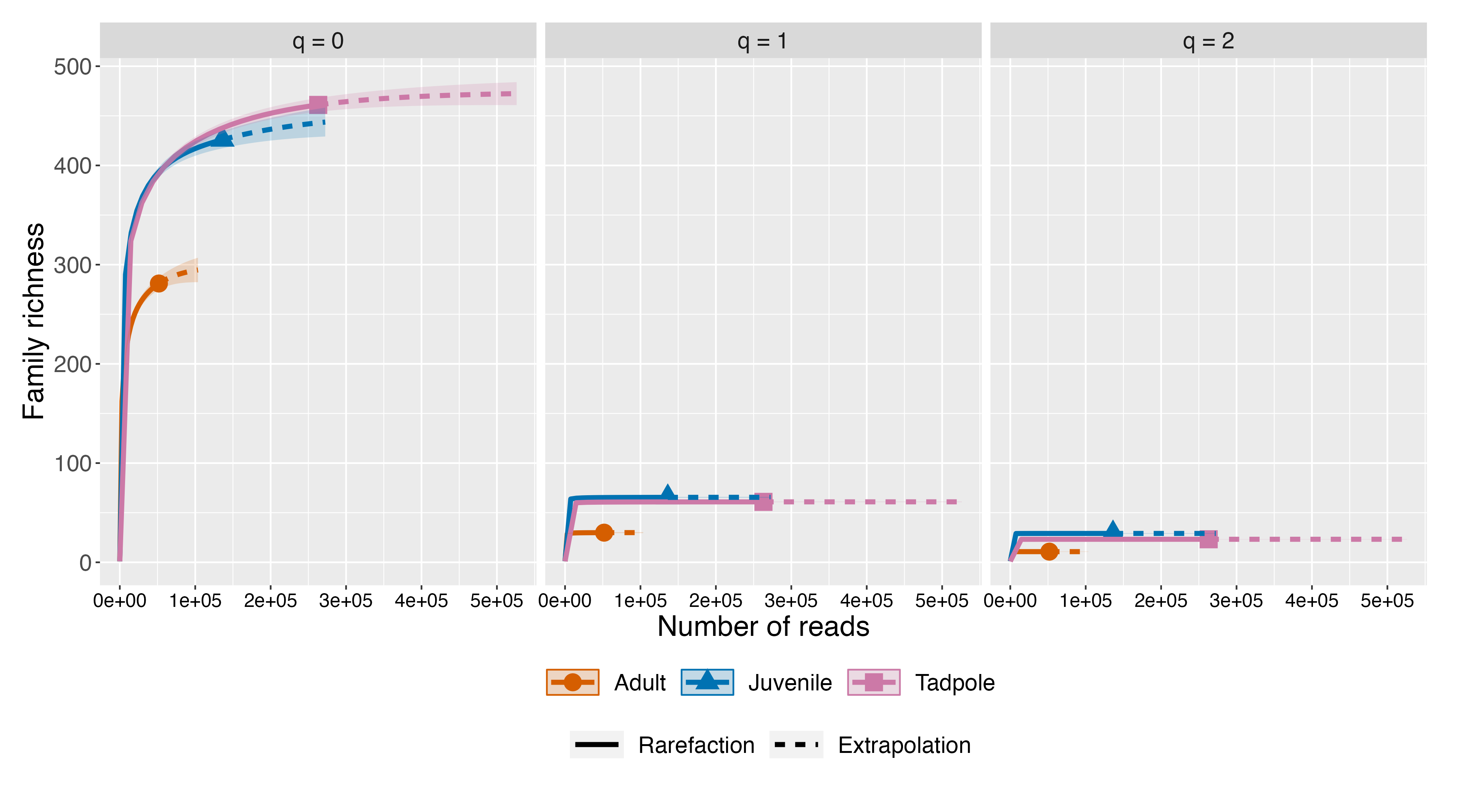


**Figure 3**. Venn diagram illustrating the number of genera with known anti-*Bd* properties shared between *Bd*+ and *Bd*- frogs.


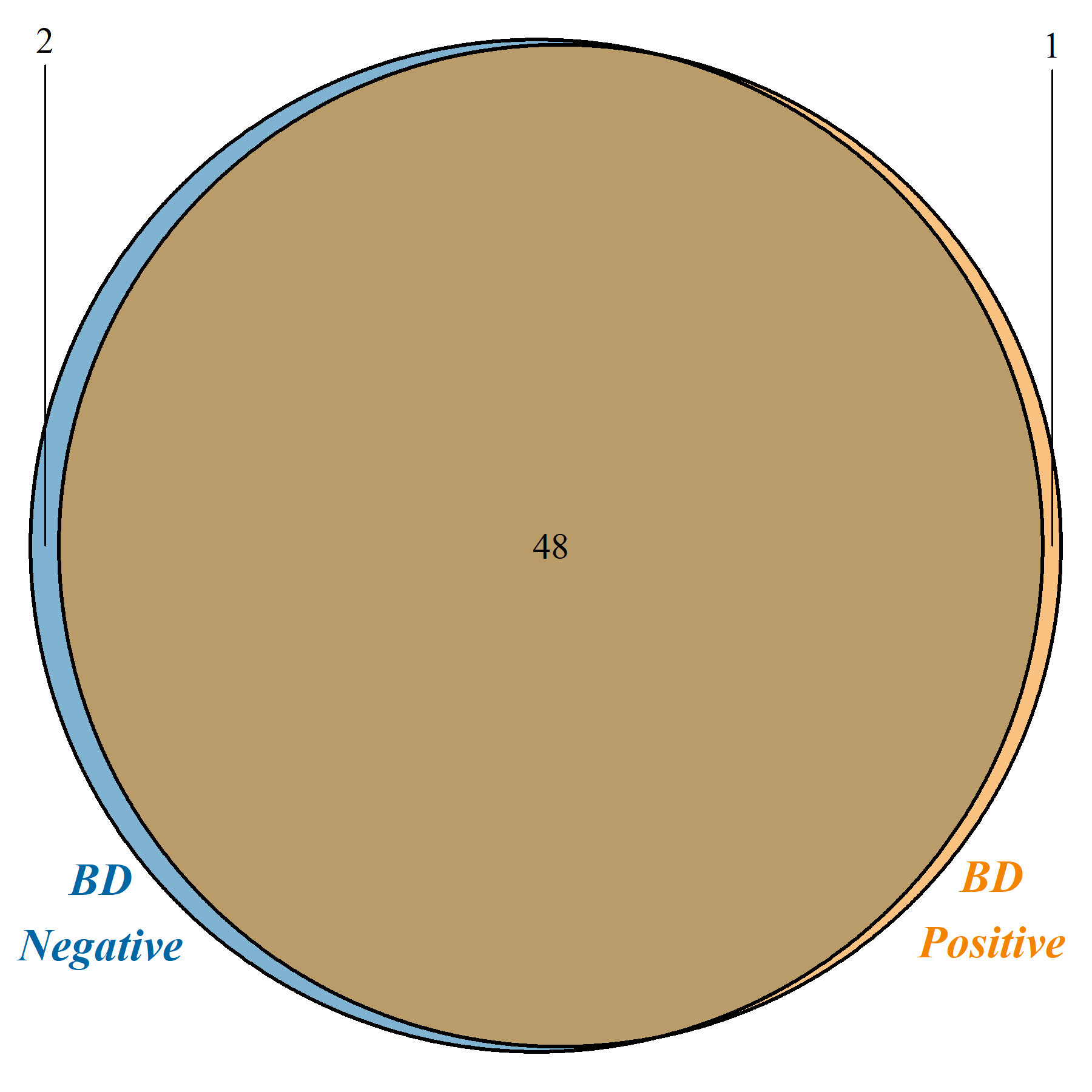

Supplement: Supplementary file 1 — Data S1 [file ECE3-14-e11249-s001.doc]
